# Late‐life onset psychotic symptoms and incident cognitive impairment in people without dementia: Modification by genetic risk for Alzheimer's disease

**DOI:** 10.1002/trc2.12386

**Published:** 2023-04-30

**Authors:** Byron Creese, Ryan Arathimos, Dag Aarsland, Clive Ballard, Helen Brooker, Adam Hampshire, Anne Corbett, Zahinoor Ismail

**Affiliations:** ^1^ Department of Clinical and Biomedical Sciences Faculty of Health and Life Sciences University of Exeter Exeter UK; ^2^ Social Genetic and Developmental Psychiatry Centre Institute of Psychiatry, Psychology and Neuroscience King's College London London UK; ^3^ Department of Old Age Psychiatry Institute of Psychiatry Psychology and Neuroscience King's College London London UK; ^4^ Centre for Age‐Related Medicine Stavanger University Hospital Stavanger Norway; ^5^ Department of Health and Community Sciences Faculty of Health and Life Sciences University of Exeter Exeter UK; ^6^ Department of Brain Sciences Faculty of Medicine Imperial College London London UK; ^7^ Department of Health and Community Sciences Faculty of Health and Life Sciences University of Exeter UK; ^8^ Departments of Psychiatry Clinical Neurosciences, and Community Health Sciences Hotchkiss Brain Institute and O'Brien Institute for Public Health University of Calgary Calgary Alberta Canada; ^9^ Department of Health and Community Sciences, Faculty of Health and Life Sciences University of Exeter Exeter UK

**Keywords:** *APOE*, cognition, mild behavioral impairment, neuropsychiatric symptoms, psychosis

## Abstract

**Introduction:**

Late‐life onset psychosis is associated with faster progression to dementia in cognitively normal people, but little is known about its relationship with cognitive impairment in advance of dementia.

**Methods:**

Clinical and genetic data from 2750 people ≥50 years of age without dementia were analyzed. Incident cognitive impairment was operationalized using the Informant Questionnaire on Cognitive Decline in the Elderly (IQCODE) and psychosis was rated using the Mild Behavioral Impairment Checklist (henceforth MBI‐psychosis). The whole sample was analyzed before stratification on apolipoprotein E (*APOE*) ε4 status.

**Results:**

In Cox proportional hazards models, MBI‐psychosis had a higher hazard for cognitive impairment relative to the No Psychosis group (hazard ratio [HR]: 3.6, 95% confidence interval [CI]: 2.2–6, *p* < 0.0001). The hazard for MBI‐psychosis was higher in *APOE* ε4 carriers and there was an interaction between the two (HR for interaction: 3.4, 95% CI: 1.2–9.8, *p* = 0.02).

**Discussion:**

Psychosis assessment in the MBI framework is associated with incident cognitive impairment in advance of dementia. These symptoms may be particularly important in the context of *APOE* genotype.

## 1 BACKGROUND

Broadly comprising visual hallucinations, paranoid delusions, and misidentifications, psychosis occurs in around 40% of people with Alzheimer's disease (AD) dementia, and most people with dementia with Lewy bodies and Parkinson's disease dementia.^1^ Although much of the research literature in cognitive aging has focused on symptoms that occur in the context of established dementia, psychotic symptoms do occur in later life in people without dementia. Recognizing the significance of such symptoms, the International Psychogeriatric Association (IPA) recently revised their criteria for psychosis in neurocognitive disorders to include symptoms occurring in mild neurocognitive disorders (i.e., in advance of dementia in the mild cognitive impairment [MCI] stage).^2^ Moreover, new research criteria for psychosis in AD from the Alzheimer's Association International Society to Advance Alzheimer's Research and Treatment (ISTAART) Neuropsychiatric Syndrome Professional Interest Area stipulate that psychosis due to underlying neurodegenerative disease can occur even earlier, in normal cognition.^3^ These criteria build upon the development of the mild behavioral impairment (MBI) framework. This framework describes a spectrum of neuropsychiatric symptoms (NPS) across domains of apathy, mood/anxiety, impulse dyscontrol, social inappropriateness, and psychosis that can present as an early manifestation of neurodegenerative disease.^4^, ^5^ To qualify as MBI, symptoms must start in later life (≥50 years), persist at least intermittently for ≥6 months, and not be better explained by another medical condition (e.g., psychotic depression or late‐onset schizophrenia). Domains do overlap in individuals, with comorbid agitation being highlighted as particularly relevant in the recent ISTAART criteria.^3^ As a broad syndrome—comprising any of the five aforementioned domains—MBI is linked to cognitive decline and progression to dementia but research on risk attributable to individual MBI domains is still in a nascent phase.^6^, ^7^, ^8^, ^9^, ^10^

**RESEARCH IN CONTEXT**

1. Systematic Review: The authors reviewed the peer‐reviewed literature and drew upon relevant conference abstracts. Although there is evidence that psychosis in the context of clinical psychiatric conditions is associated with dementia in cognitively normal people, less is known about syndromes that occur outside of a psychiatric diagnosis. Mild behavioral impairment (MBI) provides a validated context for such studies.
2. Interpretation: Our findings show that psychosis is a risk factor for incident cognitive impairment in advance of dementia, particularly in people with at least one apolipoprotein E (*APOE*) ε4 allele. New‐onset, relatively mild symptoms in later life could warrant a cognitive evaluation in clinic.
3. Future Directions: The findings provide a rationale for future studies to examine psychotic symptoms assessed in the MBI framework in more detail, including evaluation of biomarkers to confirm etiology and longitudinal evaluation of clinical outcomes.

**HIGHLIGHTS**

- Late‐life psychotic symptoms were assessed in cognitively normal people. Psychosis symptoms conferred a higher hazard for cognitive impairment.
- The hazard for cognitive impairment associated with psychosis was higher in apolipoprotein E (*APOE*)‐ε4 than non‐carriers.
- This highlights the importance of late‐life psychotic symptoms in cognitive aging.

Late‐life onset psychotic symptoms in advance of dementia are uncommon but do appear to have clinically significant consequences. In MCI, delusions occur in less than 10% of people, whereas hallucinations are present in 1%–2%. In cognitively normal older individuals, psychosis prevalence when assessed in the MBI framework is ≈3% but has been reported as high as 10%–12% (henceforth MBI‐psychosis).^11^, ^12^, ^13^ In people with normal cognition, most studies are focused on clinically defined groups of patients with psychotic disorders (e.g., delusional disorder, late‐onset schizophrenia, or very‐late‐onset schizophrenia‐like psychosis). Overall, these tend to show an increased risk of dementia; however, studies on individuals whose symptoms are not attributable to another clinical disorder are scarce.^14^, ^15^, ^16^, ^17^ What few data that are available align with the findings in clinically defined samples and suggest that psychosis confers the highest risk for dementia of all neuropsychiatric syndromes in cognitively normal people.^18^, ^19^

With an intensifying need to identify early markers of neurodegenerative disease it is imperative that further characterization of a low frequency, but seemingly high‐risk, syndrome‐like psychosis is carried out in cognitively normal samples. Key avenues relate to the impact of psychosis on functional outcomes and identification of the most clinically relevant symptom profiles. In addition, methodologically, there are two observations from research to date that have informed the present study. The first is that psychosis tends to be treated as a composite of delusions and hallucinations, and the second is that co‐morbid NPS are not always controlled for. On the first point, delusions and hallucinations may have distinct neurobiological correlates, providing a rationale to examine clinical correlates independently.^20^ On the second point, given that symptoms like agitation overlap with psychosis, controlling for comorbid NPSs will be important to determine the principal drivers of incident cognitive decline.^21^

In the present study, we examined (1) the relationship between MBI‐psychosis and incident cognitive impairment operationalized by the Informant Questionnaire on Cognitive Decline in the Elderly (IQCODE); and (2) whether this relationship was modified by gender and genetic risk for AD. This analysis complements emerging computerized testing data because the IQCODE has a reference period of 10 years, is not confounded by education or intelligence, and captures cognitive impairments that can give a better picture of the impact of symptoms on everyday functioning.

## 2 METHODS

### 2.1 Data source and participant selection

Data used in this analysis are from PROTECT (www.protectstudy.org.uk; Research Ethics Committee reference number 13/LO/1578), a UK‐based online study that examines lifestyle, mental health, and genetic risk factors for cognitive aging. PROTECT was launched in 2015 from King's College, London, and moved to the University of Exeter, UK, in 2017. The data analyzed here are taken from a January 2022 data freeze. Participants volunteered to take part in response to a national publicity drive that initially started in late 2015 and included radio and local publicity. Recruitment has been open continuously since its launch. Written informed consent from participants is obtained online. Participants may nominate a study partner who is required to know the participant well for at least 10 years. The study partner answers two questionnaires, the Mild Behavioral Impairment Checklist (MBI‐C) and IQCODE, which assess NPS and cognitive impairment, respectively (see Sections 2.3 and 2.4) . All assessments are completed online and annually (within a 6‐month window of the enrollment anniversary date). Participants can volunteer to provide a saliva sample by mail, which is used for genotyping. Upon enrollment, participants confirm that they do not have a diagnosis of dementia, do have access to a computer and the internet, are age 50 years or older, and are able to read and write English.

**FIGURE 1:**
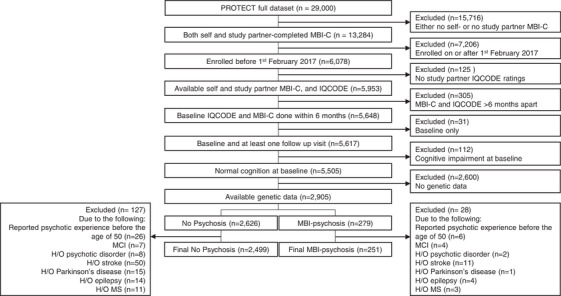
CONSORT chart showing participant selection.

All participants who nominated a study partner, had available genetic data, and were cognitively normal were considered for analysis. Normal cognition was defined as having baseline IQCODE <3.6 (see Section 2.3 below for more detail) and answering “no” to the question “Have you ever received a diagnosis from a medical professional of mild cognitive impairment (MCI)?” Only individuals who enrolled on or before February 1, 2017, were included in the analysis, to ensure that per protocol follow‐up time was at least 5 years (an appropriate time for a cognitively normal, relatively young sample). Other exclusions applied are detailed below and in the CONSORT diagram in Figure 1.

### 2.2 Demographic and clinical history

Age, gender, education level, ethnicity, medical history, and the presence of any hearing or vision problems at baseline were recorded. Hearing and vision problems were ascertained by the questions: “Do you have problems with your hearing?” and “Do you have any visual impairment that requires you to wear glasses/contact lenses?” Previous diagnoses of the following conditions were obtained by self‐report: hypertension, stroke, heart disease/attack/angina, diabetes, MCI, Parkinson's disease, high cholesterol, hypothyroidism, hyperthyroidism, arthritis, Huntington's disease, current cancer, remitted cancer, osteoporosis, asthma, epilepsy, motor neuron disease, multiple sclerosis, Paget's disease, deep vein thrombosis, HIV/AIDS, hepatitis C. Similarly, self‐reported history of diagnosis of any of the following psychiatric conditions was also recorded: depression, mania/bipolar depression, anxiety/generalized anxiety disorder, social anxiety disorder, agoraphobia, panic attacks, obsessive compulsive disorder, anorexia nervosa, bulimia nervosa, binge eating, schizophrenia, any other type of psychotic illness, personality disorder, autism spectrum disorder, attention‐deficit/hyperactivity disorder. The presence of schizophrenia or any other psychotic disorder was coded separately, whereas all other mental health conditions were coded collectively as “history of a mental health condition.” Finally, four questions were asked pertaining to the presence of psychotic experiences during the participant's lifetime, covering hallucinations (visual and auditory) and delusions (the full questions are listed in the Supplementary Material). Following these, participants were also asked at what age they first had such experiences. These questions provide an additional screening measure to identify people with early life psychotic experiences that would not be captured by the questions relating to diagnosed mental health conditions.

### 2.3 Primary outcome: Incident cognitive impairment

Incident cognitive impairment was operationalized as progression from a study partner‐rated IQCODE of <3.6 (at baseline) to a score of >3.6 (note IQCODE = 3.6 is an impossible value). The rationale for this choice of outcome is explained below. The IQCODE is a 16‐item scale where respondents are asked to rate change in cognitive ability across a range of everyday activities over the last 10 years on a scale of 1 (much better) to 5 (much worse), with 3 representing no change. The full list of questions is included in the Supplement. The final IQCODE score is obtained by taking the mean of the 16 items. Various cut points are used to capture cognitive impairment in community samples, typically ranging from 3.3 to 3.6.^22^ For this study, IQCODE >3.3 and >3.6 were considered. To inform choice of cognitive endpoint, we tested the association of each score cross‐sectionally at baseline with apolipoprotein E (*APOE*) ε4 status in the whole PROTECT sample (*N* = 6151). In logistic regression analysis with each IQCODE cut point as the binary dependent variable, carrying two copies of the *APOE* ε4 allele was associated with worse baseline cognition relative to no copies. There was a modestly higher odds ratio (OR) for the 3.6 cut point in PROTECT, so we selected this cut‐point as the outcome (>3.6: OR = 2.21, 95% confidence interval [CI]: 1.22–3.71, *p* = 0.005; >3.3: OR = 1.6, 95% CI: 1.12–2.24, *p* = 0.008). For context, a mean score of >3.6 can be reached by rating “much worse” (i.e., 5) on at least 5 of 16 items or “a bit worse” (i.e., 4) on 10 of 16 items (assuming all others are rated as “no change”). Follow‐up times were calculated from baseline to date of outcome, censoring, or 5 years for those who remained cognitively unimpaired throughout.

Online delivery of the IQCODE has been shown to correlate strongly with the traditional paper version.^23^

### 2.4 MBI‐psychosis

Psychotic symptom status was ascertained from the Mild Behavioral Impairment Checklist (MBI‐C),^24^ which has been validated for online use.^11^, ^25^ Both participants and their study partners provided ratings. A total of 33 questions capture symptoms in five domains (mood, apathy, impulse dyscontrol, social inappropriateness, and psychosis). Each item is first rated as present or absent; if rated present, the severity of the item is then scored on a scale of 1 to 3.^26^

To reflect MBI diagnostic criteria, the MBI‐C is prefixed with the following instructions to participants (with wording amended accordingly for study partner ratings): “We would like to know if there have been any subtle changes in your behavior such as changed interest in activities, altered mood, or impulsive behavior.” Answer options for the questions are as follows: “Yes: the behavior has been present for at least 6 months (continuously, or on and off) and is a change from your longstanding pattern of behavior. No: behavior not present, or present for less than 6 months, no change from usual behavior. Mild: noticeable, but not a significant change. Moderate: significant, but not a dramatic change. Severe: very marked or prominent, a dramatic change.”

There are five MBI‐C questions pertaining to psychosis; three questions cover delusion‐type experiences, which includes overvalued ideas (paranoid, harm, and grandiose‐type), and two cover hallucinations (visual and auditory). MBI‐C ratings were completed within 6 months of the initial IQCODE assessment. Ratings of participants and study partners had to be within 6 months of each other (Figure 1). Based on these ratings, two groups were created: MBI‐psychosis and No Psychosis. Participants were classified as MBI‐psychosis if they or their study partner rated any of the five psychosis items as present at their first visit. Participants were coded as No Psychosis if they scored zero on all five items on both participant and study partner ratings. To reflect MBI diagnostic criteria (which stipulate symptoms should not be attributable to another medical condition), participants with Parkinson's disease, epilepsy, multiple sclerosis, or history of stroke were excluded (see Figure 1). Moreover, although the MBI‐C instructions state that symptoms should be new onset, there were 33 participants who reported having a psychotic experience before the age of 50 according to the four lifetime psychotic experience questions described in Section 2.2. We excluded these participants to ensure only late‐life onset psychosis cases were in our sample.

### 2.5 Computerized cognitive test scores

At baseline, participants completed a battery of computerized cognitive tests comprising paired associate learning, digit span, self‐ordered search, and verbal reasoning.^27^ We constructed a general cognitive composite score using scores across each of these four tests as described previously.^28^ Briefly, we did this by computing the first unrotated principal component of the cognitive test battery. This composite variable was used as a covariate to adjust for cognitive ability at baseline.

### 2.6 Genotype quality control, *APOE* determination, and polygenic risk score calculation

Standard genotype quality control (QC) steps were followed before *APOE* genotypes were determined and polygenic risk scores (PRS) for AD were calculated. A detailed description of genotyping, QC, and imputation is provided in the Supplement (described previously^28^). Genotype data (Illumina Global Screening Array, GSA) were available for 9146 PROTECT study participants. Individual‐level QC steps included call‐rate (98%) filtering, relatedness, excess heterozygosity, and gender mismatch. Individuals not of European ancestry were excluded. These steps led to the removal of 790 people. Variant‐level QC included call‐rate (98%) and Hardy‐Weinberg deviation (*p* < 0.00001). Genotypes were imputed to the 1000 Genomes European reference panel using the Michigan imputation server and genotype phasing using Eagle. Variants were restricted to single nucleotide polymorphisms (SNPs) only, with a minor allele frequency (MAF) >0.001. An absolute cutoff of 0.7 was applied to the imputation quality of variants (*R^2^* as reported by the Michigan imputation server). The number of variants remaining after QC was 9,415,055.

*APOE* genotype was determined from SNPs rs429358 and rs7412, which were genotyped directly on the GSA array. A PRS for AD was calculated to capture non‐*APOE* genetic risk.

The AD PRS was calculated using International Genomics of Alzheimer's Project (IGAP) AD genome‐wide association study (GWAS) summary statistics, using PRSice v2.2.12.^29^, ^30^ Briefly, the PRS is generated by calculating the effect size‐weighted sum of the number of risk alleles carried at each SNP by an individual. All available SNPs (83,540) from the IGAP study were used to calculate the PRS. This PRS was associated with cognitive performance on computerized testing in a sample that overlaps with the current one.^28^ AD PRS tertiles were generated in the whole sample before the phenotype exclusions described in Figure 1.

### 2.7 Statistical analysis

We compared baseline demographic, clinical, and genetic variables between the MBI‐psychosis and No Psychosis groups using *t*‐tests (or Mann‐Whitney test for non‐parametric data) or Fisher's exact test.

We compared progression to cognitive impairment (to IQCODE >3.6) between the MBI groups using reverse Kaplan‐Meier (KM) cumulative event curves and the log‐rank test over a maximum follow‐up period of 5 years. Following this, we fitted Cox proportional hazards models to the data and generated hazard ratios (HRs) to test the difference in rates of incident cognitive impairment in the MBI‐psychosis group relative to the No Psychosis group. Additional covariates were gender, age, history of any mental health condition, and *APOE* ε4 status.

We then stratified the sample on *APOE* ε4 status, and then separately on gender (informed by previous research showing interactions between gender and psychosis on cognitive decline^31^). We then explored progression to incident cognitive impairment for MBI‐psychosis versus No Psychosis in each *APOE* and gender stratum. Finally, we fitted interaction terms to the models (MBI‐psychosis*APOE ε4 then MBI‐psychosis*gender) . To explore the role of non‐*APOE* genetic variation, we generated HRs for MBI‐psychosis status in each AD PRS tertile. Covariates remained as above, but to evaluate the contribution of other non‐psychosis MBI items, we entered MBI‐C total score excluding the five psychosis items as a covariate in a second adjusted model. Finally, we examined the influence of objectively measured cognitive performance at baseline by including the cognitive test composite as a covariate.^28^

We completed all analyses in R version 4.1.3 using the *survminer* package for cumulative event plots and the *survival* package for Cox proportional hazards models. We tested proportional hazards assumptions and confirmed them using the *cox.zph* function.

## 3 RESULTS

### 3.1 Participant demographics and clinical characteristics

After the exclusions detailed in Figure 1, a total of 2750 participants were included in the final analytical sample (mean age 64 ± 6.8; 2165 [74%] women). There were 2499 participants classified as having No Psychosis and 251 in the MBI‐psychosis group (Table 1). The 251 people with MBI‐psychosis comprised 77 with self‐rated but not proxy‐rated symptoms; 156 with proxy‐rated but not self‐rated symptoms; and 18 with both proxy‐ and self‐rated symptoms. This is in line with our previous publication using a larger sample of the PROTECT cohort, which included all cases in this study.^11^ There were no statistically significant differences for the MBI‐psychosis and No Psychosis groups on any demographic variables, but a numerically higher proportion of individuals in the No Psychosis group had undergraduate degrees (37% vs 29%). The MBI‐psychosis group performed worse on baseline computerized testing (MBI‐psychosis mean score: 0.01; No Psychosis mean score: 0.21; *t*‐test of the difference: t = 2.63, df = 303, *p* = 0.009). In terms of self‐reported ethnicity (note this may differ from genetically determined ancestry), virtually all participants reported being White (British—including English, Scottish and Welsh, Irish, other European or non‐European), with five reporting being of mixed‐race background.

**TABLE 1:** Baseline sample characteristics by MBI‐psychosis status

	No Psychosis		MBI Psychosis	
	2499		251	
***N***	Mean	SD	Mean	SD
**Age**	64	6.8	63	7.0
**Gender**	*n*	%	*n*	%
Male	650	26	66	26
Female	1849	74	185	74
	Median	IQR	Median	IQR
**Proxy‐rated MBI‐C total score excluding psychosis items (median, IQR)**	1	4	6	9
**Self‐rated MBI‐C total score excluding psychosis items (median, IQR)**	0	2	3	9
	*n*	%	*n*	%
**Delusions** **^a^**	‐	‐	234	93
**Hallucinations** **^a^**	‐	‐	20	8
**Number of *APOE* ε4 alleles**	*n*	%	*n*	%
0	1733	69	160	64
1	702	28	85	34
2	64	3	6	2
**Education**	*n*	%	*n*	%
Left school at 16	290	12	39	16
Left school at 18	290	12	29	12
Vocational qualification	467	19	54	22
Undergraduate degree	923	37	72	29
Post‐graduate or doctoral degree	529	21	57	23
**Ethnicity** **^b^**	*n*	%	*n*	%
White	2494	100	251	100
Mixed race (White and Black African)	2	0	0	0
Any other mixed ethnicity^c^	3	0	0	0
**Medical history** **^e^**	*n*	%	*n*	%
Wears glasses or contact lenses	2,390	96	232	92
Hearing problem	723	29	77	31
Any mental health condition^f^	873	35	115	46
Hypertension	696	28	81	32
Heart disease	150	6	20	8
Diabetes	95	4	12	5
High cholesterol	657	26	64	25
Hypothyroidism	176	7	21	8
Hyperthyroidism	50	2	3	1
Arthritis	523	21	59	24
Cancer (current)	50	2	4	2
Cancer (full remission)	207	8	29	12
Osteoporosis	154	6	17	7
Asthma	207	8	27	11
Paget's disease	1	0	0	0
Deep vein thrombosis	32	1	2	1
Hepatitis C	2	0	0	0

Abbreviations: MBI, mild behavioral impairment; MBI‐C, Mild Behavioral Impairment Checklist.

^a^ Does not sum to 235 because three participants had both delusions and hallucinations.

^b^ Refers to self‐reported ethnicity not genetically determined ancestry. Only non‐zero frequency items are presented but the following options were available: White: English/Welsh/Scottish/Northern Irish/British; White: Irish; White: Gypsy or Irish Traveler; White: European; White: Non‐European; Mixed: White and Black Caribbean; Mixed: White and Black African; Mixed: White and Asian; Mixed: Any other Mixed/Multiple ethnic background; Asian/Asian British: Indian; Asian/Asian British: Pakistani; Asian/Asian British: Bangladeshi; Asian/Asian British: Chinese; Asian/Asian British: Any other Asian background; Black/African/Caribbean/Black British: African; Black/African/Caribbean/Black British: Caribbean; Any other Black/African/Caribbean background; Other ethnic group: Arab; Any other ethnic group.

^c^ White includes any of the “White” options listed in footnote a.

^d^ Denotes any other mixed ethnic background besides those listed in footnote b.

^e^ Only conditions rated as present by at least one person are shown (see text for full list of conditions screened for).

^f^ Excludes schizophrenia and history of any other psychotic illness.

The median proxy‐rated MBI‐C total score excluding psychosis items was 6 for MBI‐psychosis and 1 for No Psychosis (W = 131215, *p* < 0.0001). The median self‐rated MBI‐C score excluding psychosis items was 3 for MBI‐psychosis and 0 for No Psychosis (W = 201904, *p* < 0.0001). Ninety‐three percent (234) of MBI‐psychosis participants had delusions and 8% (20) had hallucinations. Only three participants experienced both symptoms. The groups differed on history of any mental health condition (MBI‐psychosis 46% vs No Psychosis 35%, Fisher's exact *p* = 0.0008), this does not include schizophrenia and other psychotic disorders, as these individuals were excluded from the analysis. All other self‐reported medical conditions were comparable between the two groups (Table 1).

### Cumulative event analysis

In the whole sample, median follow‐up time was 3.9 years (interquartile range [IQR]: 2.7 years). Although incident cognitive impairment was an uncommon event, the MBI‐psychosis group had significantly lower cognitive impairment‐free survival than the No Psychosis group (5‐year survival probability for the MBI‐psychosis group: 86% [95% CI: 79%–94%]; for No Psychosis: 94% [95% CI: 91%–96%]; log‐rank *p* < 0.0001). Thus the cumulative incidence of decline to IQCODE >3.6 was 14% [95% CI: 6%–21%] for MBI‐psychosis and 6% [95% CI: 4%–9%] for No Psychosis. The corresponding survival table for the whole sample is included in the Supplement (Table S1).

**FIGURE 2:**
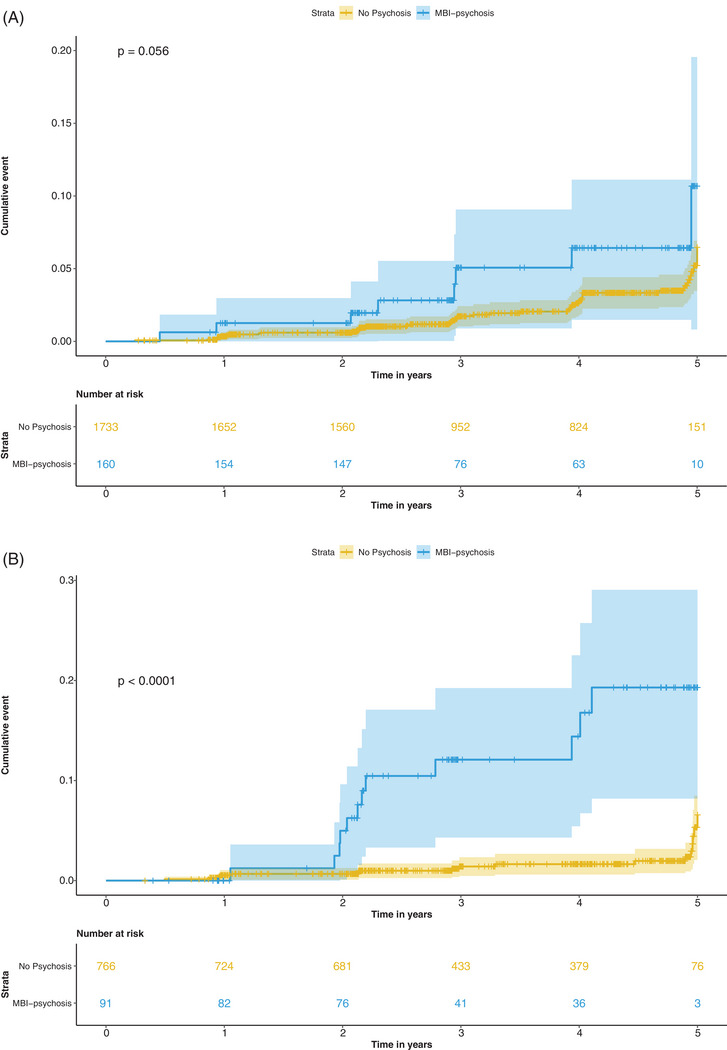
Reverse Kaplan‐Meier cumulative event curve for incident cognitive impairment over 5 years, stratified by MBI‐psychosis status in *APOE* ε4 non‐carriers (A) and carriers (B). *p*‐values are from the log‐rank test.

We then created two strata for *APOE* status (by grouping carriers of one or two alleles together due to low numbers): no ε4 alleles (MBI‐psychosis, *n* = 160; No Psychosis, *n* = 1733) and one or two ε4 alleles (‘“ε4 carrier,” MBI‐psychosis, *n* = 91; No Psychosis, *n* = 766). Figures 2A and B show the reverse KM cumulative event curves for cognitive impairment probability over 5 years for MBI‐psychosis compared with No Psychosis in *APOE* ε4 non‐carriers (A) and carriers (B). In *APOE* ε4 carriers, MBI‐psychosis had a lower survival probability relative to No Psychosis (9% [95% CI: 8%–29%] vs 6% [95% CI: 2%–10%, log rank *p* < 0.0001). Survival probability did not differ between the two groups in *APOE* ε4 non‐carriers (11% [95% CI: 1%–20%] vs 6% [95% CI: 4%–9%, log rank *p* = 0.06).

### Cox proportional hazards analysis

Full lists of regression coefficients, standard errors, and *p*‐values are included in the Supplement.

#### Whole sample

The MBI‐psychosis group had a 3.6‐fold higher incidence of cognitive impairment than No Psychosis (HR: 3.6, 95% CI: 2.2–6, *p* < 0.0001, see Figure S1 for a forest plot of adjusted HRs).

#### Stratified analyses

**FIGURE 3:**
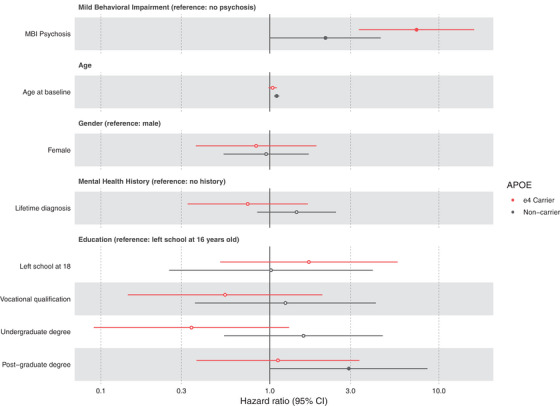
Forest plot of adjusted hazard ratios for incident cognitive impairment across all covariates, stratified by *APOE* carrier status (0 vs 1 or 2 ε4 alleles). Filled points denote statistical significance at *p* < 0.05.

Figure 3 and Figure S2 show the whole sample stratified by *APOE* status and gender, respectively.

##### Genetic risk for AD

*APOE*. There was a 2.1‐fold higher hazard for cognitive impairment in the MBI‐psychosis group relative to the No Psychosis group in *APOE* ε4 non‐carriers, but this did not reach statistical significance (95% CI: 1–4.4, *p* = 0.05). In ε4 carriers, MBI‐psychosis had a 7.3‐fold greater hazard than No Psychosis (95% CI: 3.4–16.2, *p* < 0.0001). The interaction between MBI‐psychosis and *APOE* ε4 was statistically significant (HR: 3.4, 95% CI: 1.2–9.8, *p* = 0.02, Figure 3).

AD PRS. The HR for cognitive impairment for MBI‐psychosis relative to No Psychosis was comparable across the three PRS tertiles. In the lowest tertile, there was a 3.4‐fold higher rate of cognitive impairment in MBI‐psychosis relative to No Psychosis (95% CI: 1.3–8.5, *p* = 0.01). In the middle tertile, there was a 4.4‐fold higher rate (95% CI: 2–9.6, *p* = 0.0002). In the top tertile, there was a 3‐fold higher rate (95% CI: 1.1–8.2, *p* = 0.02). There was no interaction between MBI‐psychosis status and AD PRS tertile.

A cross‐tabulation of *APOE* carrier status by AD PRS tertile showed that a significant proportion of ε4 carriers were present across the lowest, middle, and highest tertiles (24%, 33%, and 37%, respectively). Thus, the *APOE* signal is spread across the PRS strata, which may explain the difference in results between the *APOE* strata and the PRS strata. To explore this further post hoc, we examined a second AD PRS that included only those SNPs showing genome‐wide significant association with AD (i.e., 22 SNPs, including the *APOE* locus, calculated as described previously^28^). As would be expected, in the top tertile of this PRS, 76% of people carried at least one *APOE* ε4 allele compared to 13% of the middle tertile, and 1% of the bottom tertile. Here, we observed a similar interaction as for *APOE* (MBI‐psychosis*top tertile: HR: 7, 95% CI: 1.4–3.5, *p* = 0.02; MBI‐psychosis*middle tertile: HR: 3.4, 95% CI: 0.6–1.9, *p* = 0.6).

Controlling for non‐psychosis MBI domains. When controlling for other NPSs, MBI‐psychosis was no longer associated with a higher rate of incident cognitive impairment in *APOE* ε4 non‐carriers (HR: 0.9, 95% CI: 0.4–2, *p* = 0.8). In *APOE* ε4 carriers, the HR was reduced from 7.4 to 3.6 (95% CI: 1.6–8.4). The interaction between the MBI‐psychosis group and *APOE* carrier status remained statistically significant (HR: 4.2, 95% CI: 1.4–12.1, *p* = 0.009).

Controlling for baseline general cognition. When incorporating baseline computerized neuropsychological test performance, there was a slight reduction in the HR for the MBI‐psychosis*APOE ε4 interaction term but the Cis largely overlapped with the model that did not include general cognition as a covariate (HR: 3.9, 95% CI: 1.3–11.3, *p* = 0.01).

Analysis of delusions only. There were only 20 people with hallucinations; however, given that these symptoms may reflect different neurobiological substrates, we removed them and repeated the analysis (i.e., Cox proportional hazards models controlling for age, gender, education, mental health diagnosis history, and non‐psychosis MBI domains). The interaction between MBI‐psychosis group and *APOE* carrier status was statistically significant, with estimates similar to the primary analysis described earlier (HR: 3.8, 95% CI: 1.3–11.3, *p* = 0.02).

##### Gender

The hazard for cognitive impairment was 5.2‐fold higher in MBI‐psychosis relative to No Psychosis in men (95% CI: 2.2–12.4, *p* = 0.0001), and 2.9‐fold higher than No Psychosis in women (95% CI: 1.5–5.6, *p* = 0.001). The interaction did not reach statistical significance (HR: 0.6, 95% CI: 0.2–1.6, *p* = 0.3, Figure S2).

### Sensitivity analysis of the non‐genetic findings

Finally, we repeated the analysis above that does not require genetic data, that is, by including participants that were originally excluded because they did not have genetic data (Figure 1). Broadly, findings were similar when using this larger sample. There were an additional 2106 people who met criteria for inclusion to the study but who did not have genetic data available (total *N* = 4766, including the 2750 used in the main analysis described above). Post hoc, we used these data to ascertain (1) whether there were any differences in incident cognitive impairment risk between people with self‐reported and proxy‐reported symptoms; (2) whether the main findings of MBI‐psychosis and higher risk of cognitive impairment still held; and (3) whether the gender stratified analysis still held.

There were 135 people with self‐reported MBI‐psychosis but not proxy‐reported symptoms, 250 with proxy‐reported but not self‐reported symptoms, and 30 with both self and proxy‐reported symptoms. One‐hundred fifty people developed incident cognitive impairment. In Cox proportional hazards models controlling for covariates as described above, the HRs for self and proxy MBI‐psychosis relative to No Psychosis were similar (self HR: 3.9, 95% CI: 2.1–7.2, *p* = 2.2*10^−05^; proxy HR: 4.8, 95% CI: 3.2–7.4, *p* = 7.1*10^−13^). Having both symptoms was not associated with a statistically higher hazard, although Cis were wide, attendant with a sample of only 30 people being in this category (HR: 3.5, 95% CI: 0.9–14.6, *p* = 0.08). Grouping cases with any self‐ or proxy‐rated symptoms into a single MBI‐psychosis group (i.e., replicating the whole sample analysis in Section 3.3.1) yielded an HR of 4.4 for MBI‐psychosis relative to No Psychosis (95% CI: 3.1–6.4, *p* = 2.5*10^−15^).

In the gender‐stratified analysis, the hazard rate for cognitive impairment was 5.8‐fold higher (95% CI: 3.1–10.6, *p* = 1.5*10^−08^) in the MBI‐psychosis group relative to the No Psychosis group in men and 3.8‐fold higher (95% CI: 2.4–6.1, *p* = 2.83*10^−05^) than No Psychosis in women. The interaction did not reach statistical significance (HR: 0.6, 95% CI: 0.3–1.3, *p* = 0.18).

The analysis of non‐genetic findings in a sample with more than 2000 more cases available supports our main findings. It also justifies our inclusion of self‐ and proxy‐rated MBI‐psychosis symptoms together.

### 3.2 Analysis using an IQCODE cut point of ≥3.3 as the cognitive outcome

During the peer review process, we were requested to repeat the primary analysis using an IQCODE cut point of >3.3 to define the outcome of incident cognitive impairment. MBI‐psychosis was still associated with incident cognitive impairment but there was no interaction with *APOE*. Further details of this analysis are included in the Supplement.

## 4 DISCUSSION

These data add to converging evidence that highlight the importance of late‐life onset psychotic symptoms in cognitive aging. Psychotic symptoms in cognitively normal individuals were associated with a 3.6‐fold higher rate of incident cognitive impairment. Symptoms were, however, relatively uncommon, occurring in 9% of the sample (with only 0.7% experiencing hallucinations). This study confirms the impact of psychotic symptoms on cognitive abilities that affect daily life in advance of dementia, building upon studies that have examined cognitive trajectories and those that have followed individuals to the point of clinical dementia diagnosis.^18^, ^19^, ^31^

We found evidence of effect modification by *APOE* status; a significantly greater HR for incident cognitive decline in the MBI‐psychosis group was present only in individuals who carry at least one ε4 allele. This relationship was not wholly attributable to other MBI symptoms, differences in objectively measured cognition at baseline, or by people with hallucinations. That said, given only 20 people in our sample had hallucinations, we cannot draw any conclusions about different risk attributable to individual psychotic symptoms. *APOE* is a well‐established risk factor for AD and has been shown to interact with affective NPSs to influence dementia risk.^32^ Ours is the first study to show an interaction with psychosis on cognitive impairment in advance of dementia. These findings bring a new perspective to the broader relationship between *APOE* and cognitive decline by suggesting that the presence of psychotic symptoms among carriers may be of particular clinical interest. Within conventional psychiatric frameworks, older adults presenting with psychotic symptoms may not always undergo a formal cognitive assessment. However, our findings form part of an emerging literature that underscores the importance of incident cognitive decline associated with late‐life onset behavioral symptoms.^21^ It is also notable that psychosis is often an exclusion criterion for clinical trials in AD. Findings like ours may start to challenge such a blanket exclusion approach. Although the interaction with *APOE* is consistent with MBI‐psychosis being linked to neurodegeneration, we cannot make any firm conclusions about etiology. This is because *APOE* ε4 is not a fully penetrant risk factor and is not specific to AD. However, implied within our hypotheses is that, for some, both MBI‐psychosis and the cognitive endpoint of IQCODE >3.6 are sequelae of a common underlying neurodegenerative disease. In this regard, finding that one symptom of a neurodegenerative disease predicts another would be unsurprising but to definitively show this requires imaging and biomarker studies that can determine etiology.^33^, ^34^, ^35^, ^36^, ^37^, ^38^ Our findings provide a clear rationale for such studies.

We did not observe an interaction when analyzing the AD PRS (which includes *APOE* genotype) by tertile. It is important to note that 24% of the lowest tertile were *APOE* carriers, a substantial proportion even relative to the middle and high tertiles (33% and 37%, respectively). A possible explanation for this discrepancy is that the effect observed in the main analysis is *APOE* specific, and thus present to a degree across all three PRS tertiles. In addition to the two *APOE* SNPs, there are >83,500 other SNPs that were used to determine each person's AD PRS (and by extension their genetic risk tertile) and it appears that these are not clearly associated with the cognitive impairment outcome used in this study. Our post hoc analysis of a 22 SNP PRS (i.e., with far fewer non‐*APOE* SNPs to influence the score) supports this finding; we observed an interaction similar to that shown in the main *APOE* analysis. This is also in line with a number of other studies, which clearly show variation in findings according to which measure of genetic risk is used (a PRS with just a few SNPs, tens of thousands of SNPs, or *APOE* alone).^28^, ^39^, ^40^, ^41^, ^42^, ^43^ In addition, no interaction was observed when we repeated the analysis post hoc using an IQCODE cut point of 3.3; a discussion of this finding is included in the Supplement.

There was no effect modification by gender in this sample. In a recent study of cognitive trajectories based on computerized testing in a sample that overlaps with this one, the association between cognitive decline and psychosis was only present in men.^31^ It is possible that moderation by gender does not extend to the IQCODE, which reflects cognitive function assessed by decline in daily activities rather than precise measurement of specific cognitive domains. Alternatively, the nominal difference in this sample may be due to the low frequency of the cognitive outcome.

Here we found the combined delusions and hallucinations group to be the least common. This is in contrast to AD dementia, where the least common psychotic symptom group is individuals with hallucinations and no delusions.^1^ This may reflect an interesting point of difference in the presentation of psychosis in pre‐dementia samples. However, it is notable that hallucinations (whether alone or with delusions) were very uncommon; therefore, the differences in proportions may not be reliable estimates. When considering self or proxy ratings only, the proportion of people in our sample with MBI‐psychosis (self: 3%; proxy: 6%) is broadly in line with the 2.8% (95% CI: 1.7–4.7%) prevalence in other cognitively normal studies reported in a recent meta‐analysis.^13^ The combined proportion is 9% (i.e., either self‐ or proxy‐rated MBI‐psychosis) because there is very little overlap between self and proxy ratings. This estimate is higher than most (but not all^12^) other studies in cognitively normal people, and further exploration of the significance of the lack of overlap will be an important avenue for future research. We did not find any evidence of differences in rates of incident cognitive impairment associated with self‐ and proxy‐reported symptoms. We believe this is the first direct comparison of these two sets of respondents. More research into the appropriate measurement of late‐life NPSs is needed.

To our knowledge, this is the first study to evaluate the impact of psychotic symptoms assessed specifically in the MBI framework, using the MBI‐C, which utilized both participant and study partner ratings to capture symptoms fully. This has allowed us to assess new‐onset and persistent symptoms more precisely and in accordance with MBI criteria. The online nature of this study represents a strength, allowing us to reach participants with MBI who would most likely not be in contact with clinical services. However, we acknowledge that with this comes limitations. We had to rely on self‐reported clinical history to exclude individuals with a history of psychotic disorder or prior psychotic experiences. Although these measures may be subject to recall biases, it is a strength that we could rely on both a self‐reported diagnosis and self‐reported symptom‐based measures to exclude prior psychoses. This is supported by a large analysis of 157,363 UK Biobank participants in which 7803 people reported psychotic experiences but only 458 of these reported a psychotic disorder diagnosis. Conversely, in that study, 723 people reported a diagnosis of a psychotic disorder but only 63% of these endorsed psychotic experiences.^44^ Rates of psychotic experiences are generally higher in self‐report assessments than in clinical or lay interviews.^45^ This underscores the importance of using a variety of approaches to capture symptoms. As an online study, we were unable to employ interviews, but the tradeoff is a large sample size, which is important for detecting infrequent but clinically important symptoms like psychosis. In principle, medical record linkage could address the issues around self‐report, but this has significant practical barriers (including consent, costs, and data management) and should complement rather than replace self‐report. Although the IQCODE cut point of 3.6 has a sensitivity of >0.8 for detection of dementia, because of the online delivery we cannot be certain that all participants with dementia or MCI were excluded.^46^ In our sample there was an overrepresentation of women, in addition to those reporting White ethnicity and higher education. It is also notable that those with no access to computer and internet were excluded by design. All of these factors potentially biased our sample. Online studies are in many cases easier and more cost effective to conduct and it is likely their use will increase, but this must be done alongside concerted efforts to ensure equality of opportunity to participate in research. Finally, the GWAS summary statistics that were used to generate the AD PRS were calculated in people of European ancestry. This means by extension that our findings are not generalizable to people from other ancestries. This limitation reflects the need to diversify genetic studies and without this there is a limit to the translation of genetic research to benefit all. In the case of PROTECT, this should start with targeted recruitment of a more ethnoculturally diverse sample.

In summary, by reporting an increased hazard of incident cognitive impairment and an interaction with genetic risk for AD, this study emphasizes the importance of psychotic symptoms assessed in the MBI framework in cognitive aging.

## CONFLICT OF INTEREST STATEMENT

Clive Ballard has received contract grant funding from ACADIA, Lundbeck, Takeda, and Axovant pharmaceutical companies; and honoraria from Lundbeck, Lilly, Otsuka, and Orion pharmaceutical companies. Dag Aarsland has received research support and/or honoraria from Astra‐Zeneca, H. Lundbeck, Novartis Pharmaceuticals, and GE Health; and serves as a paid consultant for H. Lundbeck and Axovant. Zahinoor Ismail has received honoraria/consulting fees from Lundbeck and Otsuka, although not related to this work. Adam Hampshire is owner and director of Future Cognition Ltd., a software company that produces bespoke cognitive assessment technology and that was paid to produce cognitive tasks for PROTECT. Helen Brooker was employed by Wesnes Cognition 2011 to 2018, who provided some of the cognitive tests for PROTECT and is the director of Ecog Pro Ltd. who provide cognitive tests to PROTECT. All other authors have nothing to declare. Author disclosures are available in the Supporting Information.

## Supporting information

Supporting Information

Supporting Information
