# Supplementary material for: Late‐life onset psychotic symptoms and incident cognitive impairment in people without dementia: Modification by genetic risk for Alzheimer's disease
Source: Alzheimers Dement (N Y). 2023 Apr 30;9(2):e12386. doi: 10.1002/trc2.12386 (PMC10150165; doi:10.1002/trc2.12386)
Supplement: Supplementary file 1 — Supporting Information [file TRC2-9-e12386-s001.docx]

**Supplementary methods**

IQCODE questions:

Compared with 10 years ago how is this person at:

1. Remembering things about family and friends - eg, occupations, birthdays, addresses?

2. Remembering things that have happened recently?

3. Recalling conversations a few days later?

4. Remembering his/her address and telephone number?

5. Remembering what day and month it is?

6. Remembering where things are usually kept?

7. Remembering where to find things which have been put in a different place from usual?

8. Knowing how to work familiar machines around the house?

9. Learning to use a new gadget or machine around the house?

10. Learning new things in general?

11. Following a story in a book or on TV?

12. Making decisions on everyday matters?

13. Handling money for shopping?

14. Handling financial matters - eg, the pension, dealing with the bank?

15. Handling other everyday arithmetic problems - eg, knowing how much food to buy, knowing how long between visits from family or friends?

16. Using his/her intelligence to understand what's going on and to reason things through?

Description of additional psychotic experience questions used to screen out individuals with psychotic experiences before the age of 50:

1. Did you ever see something that wasn't really there that other people could not see? Please do not include any times when you were dreaming or half-asleep or under the influence of alcohol or drugs.
2. Did you ever hear things that other people said did not exist, like strange voices coming from inside your head talking to you or about you, or voices coming out of the air when there was no one around? Please do not include any times when you were dreaming or half-asleep or under the influence of alcohol or drugs.
3. Did you ever believe that a strange force was trying to communicate directly with you by sending special signs or signals that you could understand but that no one else could understand (for example through the radio or television)? Please do not include any times when you were dreaming or half-asleep or under the influence of alcohol or drugs.
4. Did you ever believe that that there was an unjust plot going on to harm you or to have people follow you, and which your family and friends did not believe existed? Please do not include any times when you were dreaming or half-asleep or under the influence of alcohol or drugs.
5. How old were you (approximately) when you first had one of these experiences (seeing a vision, hearing a voice, or believing that something strange was trying to communicate with you, or there was a plot against you)?

Genotyping and QC methods

Saliva samples were collected by post and DNA extracted by the National Institute for Health Research South London and the Maudsley National Health Service Biomedical Research Centre. Genotyping was done used the Illumina Global Screening Array with custom content (including directly genotyped single nucleotide polymorphisms [SNPs], rs429358 and rs7412, to determine APOE status). Genotyping was performed in three batches and the total numbers of PROTECT participants in the combined genotyped data was 9146. Iterative filtering for call rate at 98% completeness (for individuals and SNPs) resulted in the exclusion of 84 samples, after which 9062 remained. In the filtered data relatedness was estimated using KING 2.2.3, followed by extraction of a list of individuals that contained no pairs of individuals with a 1st-, 2nd- or 3rd-degree relationships. Variants with Hardy-Weinberg Equilibrium p-value < 0.00001 were excluded. Individuals whose gender estimated in plink did not match that reported by the study participants were excluded. Principal components (PCs) were calculated for the unrelated subset of the data using EIGENSOFT 6.1.4 after pruning using a window size of 1500 bases per 150 kb and an r-squared of 0.2. Variants in high LD regions and non-autosomal regions were also excluded. K-means clustering (assuming 4 distinct clusters) was used on the first two derived principal components to define a cluster of European ancestry individuals. Principal components were then recalculated for the cluster of individuals of European ancestry, with outlier individuals removed by EIGENSOFT if exceeding a sigma threshold of 30. Finally, individuals with excess heterozygosity (unusual patterns of genome-wide heterogeneity) calculated using the ibc function in plink v1.90 were excluded. The total number of individuals excluded when removing those that were either related, of non-European ancestry, of mismatched sex, outliers in the PC calculation or detected to have excess heterozygosity was 790 given an original sample size of 9062 participants. The difference between this number and the final analytical sample N is due to the optional nomination of study partners and the 1^st^ February 2017 cut-off applied.

**Supplementary Results**

**Supplementary table 1: Survival tables for the whole table, stratified by MBI-psychosis status.**

| **No Psychosis** | | | | | | |
| --- | --- | --- | --- | --- | --- | --- |
| time | n.risk | n.event | survival | std.err | lower 95% CI | upper 95% CI |
| 0 | 2499 | 0 | 1 | 0 | 1 | 1 |
| 1 | 2376 | 10 | 0.996 | 0.00129 | 0.993 | 0.998 |
| 2 | 2241 | 5 | 0.994 | 0.0016 | 0.991 | 0.997 |
| 3 | 1385 | 18 | 0.984 | 0.00287 | 0.978 | 0.989 |
| 4 | 1203 | 10 | 0.976 | 0.00374 | 0.969 | 0.983 |
| 5 | 227 | 22 | 0.935 | 0.01064 | 0.914 | 0.956 |
|  |  |  |  |  |  |  |
| **MBI-psychosis** | | | | | | |
| time | n.risk | n.event | survival | std.err | lower 95% CI | upper 95% CI |
| 0 | 251 | 0 | 1 | 0 | 1 | 1 |
| 1 | 236 | 2 | 0.992 | 0.00568 | 0.981 | 1 |
| 2 | 223 | 4 | 0.975 | 0.01028 | 0.955 | 0.995 |
| 3 | 117 | 9 | 0.924 | 0.0192 | 0.888 | 0.963 |
| 4 | 99 | 2 | 0.907 | 0.02234 | 0.865 | 0.952 |
| 5 | 13 | 3 | 0.861 | 0.03634 | 0.793 | 0.936 |


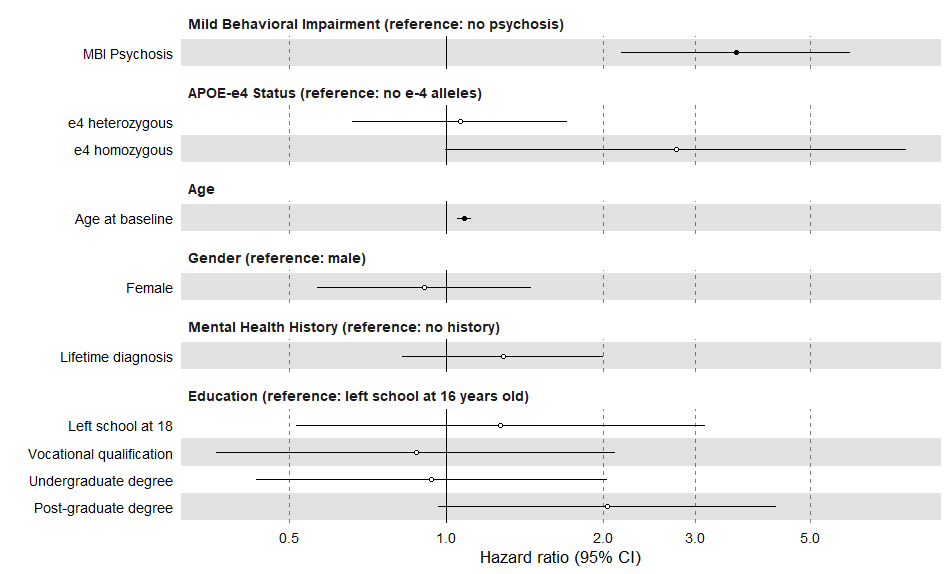


Supplementary Figure 1: Forest plot of adjusted hazard ratios for cognitive impairment across all covariates. Filled points denote statistical significance at p<0.05.


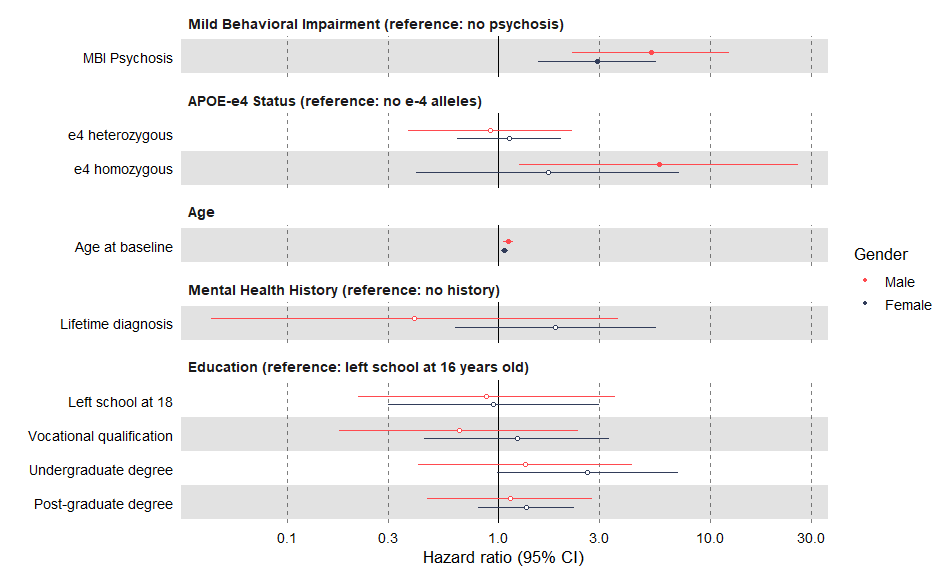


Supplementary Figure 2: Forest plot of adjusted hazard ratios for cognitive impairment across all covariates, stratified by gender. Filled points denote statistical significance at p<0.05.

Coefficients from Cox proportional hazards models

1. Whole sample

| grouping | name | beta | se | pvalue |
| --- | --- | --- | --- | --- |
| Mild Behavioral Impairment (reference: no psychosis) | MBI Psychosis | 1.280038 | 0.25912 | 7.81E-07 |
| APOE-e4 Status (reference: no e-4 alleles) | 1 or 2 alleles | 0.14953 | 0.23098 | 0.5174 |
| Age | Age at baseline | 0.078577 | 0.01554 | 4.28E-07 |
| Gender (reference: male) | Female | -0.09694 | 0.24184 | 0.6885 |
| Mental Health History (reference: no history) | Lifetime diagnosis | 0.249179 | 0.22662 | 0.2715 |
| Education (reference: left school at 16 years old) | Left school at 18 | 0.240291 | 0.46099 | 0.6022 |
| Education (reference: left school at 16 years old) | Vocational qualification | -0.13562 | 0.45075 | 0.7635 |
| Education (reference: left school at 16 years old) | Undergraduate degree | -0.06577 | 0.39542 | 0.8679 |
| Education (reference: left school at 16 years old) | Post-graduate degree | 0.711934 | 0.38119 | 0.0618 |

1. By APOE

| grouping | name | beta | se | pvalue | APOE |
| --- | --- | --- | --- | --- | --- |
| Mild Behavioral Impairment (reference: no psychosis) | MBI Psychosis | 0.761501 | 0.38402 | 0.0474 | Non-carrier |
| Age | Age at baseline | 0.092698 | 0.01844 | 4.98E-07 | Non-carrier |
| Gender (reference: male) | Female | -0.05073 | 0.2959 | 0.8639 | Non-carrier |
| Mental Health History (reference: no history) | Lifetime diagnosis | 0.37121 | 0.27382 | 0.1752 | Non-carrier |
| Education (reference: left school at 16 years old) | Left school at 18 | 0.018619 | 0.70824 | 0.979 | Non-carrier |
| Education (reference: left school at 16 years old) | Vocational qualification | 0.215598 | 0.62867 | 0.7316 | Non-carrier |
| Education (reference: left school at 16 years old) | Undergraduate degree | 0.45302 | 0.55167 | 0.4115 | Non-carrier |
| Education (reference: left school at 16 years old) | Post-graduate degree | 1.080738 | 0.54633 | 0.0479 | Non-carrier |
| Mild Behavioral Impairment (reference: no psychosis) | MBI Psychosis | 1.999344 | 0.40079 | 6.08E-07 | e4 Carrier |
| Age | Age at baseline | 0.038165 | 0.029 | 0.188 | e4 Carrier |
| Gender (reference: male) | Female | -0.18624 | 0.4193 | 0.657 | e4 Carrier |
| Mental Health History (reference: no history) | Lifetime diagnosis | -0.30128 | 0.41829 | 0.471 | e4 Carrier |
| Education (reference: left school at 16 years old) | Left school at 18 | 0.530597 | 0.61665 | 0.39 | e4 Carrier |
| Education (reference: left school at 16 years old) | Vocational qualification | -0.61085 | 0.67644 | 0.367 | e4 Carrier |
| Education (reference: left school at 16 years old) | Undergraduate degree | -1.06885 | 0.67903 | 0.115 | e4 Carrier |
| Education (reference: left school at 16 years old) | Post-graduate degree | 0.110036 | 0.56592 | 0.846 | e4 Carrier |

1. By Gender

| grouping | name | beta | se | pvalue | Gender |
| --- | --- | --- | --- | --- | --- |
| Mild Behavioral Impairment (reference: no psychosis) | MBI Psychosis | 1.658868 | 0.43657 | 0.000145 | Male |
| APOE-e4 Status (reference: no e-4 alleles) | e4 heterozygous | -0.09047 | 0.4552 | 0.842461 | Male |
| APOE-e4 Status (reference: no e-4 alleles) | e4 homozygous | 1.744323 | 0.77487 | 0.024378 | Male |
| Age | Age at baseline | 0.101003 | 0.02728 | 0.000213 | Male |
| Mental Health History (reference: no history) | Lifetime diagnosis | -0.91395 | 1.13205 | 0.419472 | Male |
| Education (reference: left school at 16 years old) | Left school at 18 | -0.13333 | 0.71465 | 0.851999 | Male |
| Education (reference: left school at 16 years old) | Vocational qualification | -0.43456 | 0.66234 | 0.511762 | Male |
| Education (reference: left school at 16 years old) | Undergraduate degree | 0.284773 | 0.59445 | 0.6319 | Male |
| Education (reference: left school at 16 years old) | Post-graduate degree | 0.122869 | 0.45848 | 0.788706 | Male |
| Mild Behavioral Impairment (reference: no psychosis) | MBI Psychosis | 1.077082 | 0.32765 | 0.001011 | Female |
| APOE-e4 Status (reference: no e-4 alleles) | e4 heterozygous | 0.115636 | 0.28791 | 0.687953 | Female |
| APOE-e4 Status (reference: no e-4 alleles) | e4 homozygous | 0.534029 | 0.72807 | 0.463263 | Female |
| Age | Age at baseline | 0.065086 | 0.01932 | 0.000756 | Female |
| Mental Health History (reference: no history) | Lifetime diagnosis | 0.617505 | 0.55894 | 0.26926 | Female |
| Education (reference: left school at 16 years old) | Left school at 18 | -0.05506 | 0.58765 | 0.925351 | Female |
| Education (reference: left school at 16 years old) | Vocational qualification | 0.19709 | 0.51195 | 0.700254 | Female |
| Education (reference: left school at 16 years old) | Undergraduate degree | 0.968106 | 0.50361 | 0.054565 | Female |
| Education (reference: left school at 16 years old) | Post-graduate degree | 0.299025 | 0.26602 | 0.260979 | Female |

Analysis using IQCODE 3.3 cut point as the cognitive outcome

During the peer review process, we were requested to repeat the primary analysis using an IQCODE cut point of >3.3 to define the outcome of incident cognitive impairment. Accordingly, as well as changing the cognitive impairment outcome we also removed any participants at baseline scoring >3.3 on the IQCODE. All other parameters were kept the same. A total of 2,467 participants were included in the analysis (less than the main analysis because of the 3.3 cut point used to remove people at baseline). After adjusting for the same covariates as described in section 3.3.1, the hazard rate for incident cognitive impairment was 2.9-fold higher in people with MBI-psychosis at baseline relative to those without (HR: 2.9, 95% CI: 2.2-3.9, p= 1.29*10^-12^). Unlike our primary analysis this risk did not differ across levels of APOE genotype (APOE ε-4 non-carriers: HR: 3.1, 95% CI: 2.2-4.4, p= 6.29*10^-10^; APOE ε-4 carriers: HR: 2.4, 95% CI: 1.4-3.9, p=0.001; p-value for the interaction=0.5).

No interaction was observed when we repeated the analysis post-hoc using an IQCODE cut point of 3.3. Intuitively, one might speculate that the specificity of the IQCODE drops with lower cut points resulting in a more heterogeneous etiology underlying the cognitive impairment status, and there is evidence from a comprehensive Cochrane review to support this[1]. Resultingly, this increased noise may have masked the APOE signal. In an online study with no in person work up, it is logical to opt for cognitive outcomes that confer the highest specificity for cognitive impairment due to dementia so we would assign more weight to our primary analysis outcome using the IQCODE cut point of 3.6.

References:

[1] Quinn TJ, Fearon P, Noel-Storr AH, Young C, McShane R, Stott DJ. Informant Questionnaire on Cognitive Decline in the Elderly (IQCODE) for the detection of dementia within community dwelling populations. Cochrane Database Syst Rev 2021. https://doi.org/10.1002/14651858.CD010079.pub3.
